# Cognitive disorders in advanced Parkinson’s disease: challenges in the diagnosis of delirium

**DOI:** 10.1186/s42466-024-00309-4

**Published:** 2024-03-14

**Authors:** Christine Daniels, Jon Rodríguez-Antigüedad, Elisabeth Jentschke, Jaime Kulisevsky, Jens Volkmann

**Affiliations:** 1 Department of Neurology, University Hospital Würzburg, Josef-Schneider-Str. 11, 97080 Würzburg, Germany; 2 Movement Disorders Unit, Sant Pau Hospital, Institut d’Investigacions Biomediques-Sant Pau, Universitat Autònoma de Barcelona, Barcelona, Spain; 3 Network Center for Biomedical Research in Neurodegenerative Diseases (CIBERNED), Madrid, Spain

**Keywords:** Psychosis, Cognition, Delirium, Parkinson’s disease

## Abstract

Parkinson’s disease (PD) is a neurodegenerative condition that is frequently associated with cognitive disorders. These can arise directly from the primary disease, or be triggered by external factors in susceptible individuals due to PD or other predisposing factors. The cognitive disorders encompass PD-associated cognitive impairment (PD-CI), delirium, PD treatment-associated cognitive side effects, cognitive non-motor fluctuations, and PD-associated psychosis. Accurate diagnosis of delirium is crucial because it often stems from an underlying disease that may be severe and require specific treatment. However, overlapping molecular mechanisms are thought to be involved in both delirium and PD, leading to similar clinical symptoms. Additionally, there is a bidirectional interaction between delirium and PD-CI, resulting in frequent concurrent processes that further complicate diagnosis. No reliable biomarker is currently available for delirium, and the diagnosis is primarily based on clinical criteria. However, the screening tools validated for diagnosing delirium in the general population have not been specifically validated for PD. Our review addresses the current challenges in the diagnosis of these cognitive disorders and highlights existing gaps within this field.

## Background

Cognitive impairment (CI) is one of the most incapacitating non-motor symptoms (NMS) in Parkinson's disease (PD), significantly affecting quality of life even during the early disease stages [1]. In the initial phases, clinical features commonly involve frontal attentional, working memory, and executive domains, while memory, language, and visuospatial deficits are typically observed during the later posterior cortical phase, predicting a more unfavorable outcome in these patients [2]. The natural course of CI in PD is heterogeneous, displaying diverse rates of progression among individuals, with some even exhibiting no progression [1]. The clinical spectrum of CI ranges from normal cognition and subjective cognitive decline (SCD) to mild cognitive impairment (PD-MCI) and PD dementia (PDD). Epidemiological studies have demonstrated that > 80% of PD patients will develop PDD within 20 years of disease onset [1].

Beyond primary PD-associated progressive CI (PD-CI), individuals with PD are frequently diagnosed with other cognitive disorders, such as delirium, PD treatment-associated cognitive side effects, cognitive non-motor fluctuations (NMF), or PD-associated psychosis, which is closely related to PD-CI [1, 3]. Among these, the correct diagnosis of delirium is particularly crucial because it often stems from a concurrent disease that may require specific treatment [4]. However, its symptoms (including inattention, disorganized thinking, fluctuating symptoms, visual hallucinations) frequently overlap with the other cognitive disorders associated with PD, leading to misdiagnosis [5]. The absence of a specific definition of delirium in PD or a reliable complementary test further complicates diagnosis. Moreover, as the disease progresses, concurrent PD-CI becomes increasingly prevalent, which poses a risk factor for delirium, PD treatment-associated cognitive side effects, cognitive NMF, and PD-associated psychosis, resulting in a common scenario of overlapping syndromes [6].

In this review, we aim to address the current challenges in the diagnosis of these cognitive disorders and highlight existing gaps within this field.

## Cognitive disorders in PD

### PD-associated cognitive impairment

PD-CI is one of the most debilitating NMS in PD [1]. SCD and PD-MCI are often present during the initial disease stages; as the disease progresses, PDD becomes more prevalent [1]. Nevertheless, the progression of PD-CI varies significantly among individuals.

The diagnosis of both PD-MCI and PDD, defined by the Movement Disorders Society (MDS) criteria, requires: (1) progressive cognitive decline in an individual with PD that does not interfere or interferes significantly with functional independence, respectively; and (2) the exclusion of other potential causes of CI, such as medication side effects, vascular disease, delirium, or other mental illnesses [7, 8]. Two levels of certainty have been defined for PD-MCI and PDD (Table 1): Level I necessitates an altered screening test, such as the PD-Cognitive Rating Scale, Montreal Cognitive Assessment, or Mattis Dementia Rating Scale; Level II requires impairment on at least two out of ten neuropsychological tests, including two tests of five different domains (attention and working memory, executive, language, memory, and visuospatial) [7, 9]. It is worth noting that since 2015, the MDS diagnostic criteria for PD allow the inclusion of early-onset dementia, overlapping with the dementia with Lewy bodies (DLB) criteria [10, 11]. This highlights the unclear boundaries between these two neuronal synucleinopathies.

**Table 1:** Updated clinical criteria for the diagnosis of PD-MCI and PDD

	PD-MCI (according to [7])	Probable PDD (according to [8])
Inclusion/exclusion criteria	Diagnosis of idiopathic PD Gradual cognitive decline (reported by patient, caregiver or clinician) Deficits at neuropsychological testing Deficits not significantly interfering with functional independence Absence of dementia or other explanations for cognitive deficits	Diagnosis of idiopathic PD Dementia syndrome with insidious onset and slow progression (cognitive impairment in more than one cognitive domain; decline from premorbid level, severe enough to impair daily life) Typical profile of neuropsychological deficits (impairment in at least two of the four core cognitive domains: attention, executive functions, visuo-spatial functions, memory) Absence of other explanations for cognitive deficits (acute confusion due to systemic diseases/abnormalities or drug intoxication; major depression; probable vascular dementia)
Specific guidelines for operationalization	According to [7]	According to [9]
Certainty level I	Abbreviated assessment (e.g. global cognitive scale, limited battery of neuropsychological tests)	Abbreviated assessment: MMSE, Pill Questionnaire, Month reversed (or Seven backwards), lexical fluency (or Clock drawing), MMSE pentagons, 3-Word recall
To be fulfilled	Abnormal global score or one abnormal* test in two domains	MMSE < 26, impairment in more than one of the tasks: month reversed (or seven backwards), lexical fluency (or clock drawing), MMSE pentagons, 3-word recall
Certainty level II	Comprehensive neuropsychological testing of each of the following five domains (minimum two tests for each domain): Attention/working memory Executive functions Language Memory Visuospatial functions	Comprehensive neuropsychological testing: global cognitive efficiency, executive functions, memory, language, visuospatial skills, neuropsychiatric features (apathy, depression, visual hallucinations, psychosis)
To be fulfilled	≥ two abnormal* tests in one domain or one abnormal* test in two domains, whole number of tests not explicitly specified	Use of age- and education-based normal values; impairment in at least two of the four core cognitive domains: attention, executive functions, visuo-spatial functions, memory; behavioral features not mandatory

*MMSE* Mini-Mental State Examination, *PD* Parkinson’s disease, *PDD* Parkinson’s disease dementia, *PD-MCI* Parkinson’s disease–mild cognitive impairment

*Abnormal corresponds to z-score − 1 to − 2 (not explicitly specified) or decline on serial neuropsychological assessments or decline from estimated premorbid functioning

PD-CI arises because of the underlying pathogenesis of PD and associated dopamine and other neurotransmitter deficits plus widespread cortico-subcortical alpha-synuclein and beta-amyloid and tau pathology [12–14]. Initially, degeneration of the nigrostriatal and mesolimbic dopaminergic pathways, along with noradrenergic output cells from the nucleus coeruleus, is considered responsible for the typical fronto-subcortical syndrome [12]. As the disease progresses, there is a severe loss of the cholinergic projections from the nucleus basalis of Meynert to the medial temporal lobe, amygdala, frontoparietal cortex, and temporoparietal association areas; this leads to posterior cortical dysfunction, which is a stronger predictor of PDD [15–17]. However, neurotransmission system dysfunction and misfolded proteins alone do not explain the pathophysiology of PD-CI; additional mechanisms are likely to be involved, including synaptic dysfunction, neuroinflammation, mitochondrial dysfunction, microglial and astroglial changes, genetics, epigenetics, adenosine receptor activation, or cerebral network dysfunction [1].

### Delirium

Despite several changes in criteria in recent decades, the Diagnostic and Statistical Manual of Mental Disorders, fifth edition (DSM-5) defines delirium as an acute-onset impairment of attention, consciousness, and cognitive performance with fluctuating characteristics over time (Table 2) [18]. Two subtypes of delirium are recognized: hyperactive delirium with agitated state represents 25% of cases, whereas hypoactive delirium with decreased levels of consciousness represents 75% of cases [19].

**Table 2:** Updated clinical criteria for the diagnosis of delirium according to DSM-5 [18]

A
Disturbance in attention and awareness
B
Develops over a short period of time/change from baseline attention and awareness/fluctuating in severity during the course of the day
C
Additional disturbance in cognition (e.g., memory, orientation, language, visuospatial ability, perception)
D
A + B not better explained by another preexisting or evolving neurocognitive disorder/no severely reduced level of arousal, such as coma
E
Evidence for precipitating medical factors or multiple etiologies

Delirium is conceptualized as cerebral dysfunction precipitated by various baseline predisposing factors (aging, CI, frailty, sensory impairment) and acute triggering factors (drugs, electrolyte and glucose metabolism disorders, uremia, surgery) [4, 19, 20]. The presence of multiple predisposing factors reduces the number of triggering factors required to induce delirium. The exact molecular pathways that cause different triggers to converge into a similar syndrome are not yet fully understood. Three main mechanisms are believed to be involved its pathophysiology. (1) The brain’s normal functioning relies on significant amounts of energy, and brain metabolic insufficiency—either from oxygen or glucose deficiency—can lead to delirium in various scenarios. (2) Inflammation is a known trigger of delirium and several circulating cytokines are found in patients with delirium, which may have a significant effect in the nervous system. (3) Several deficits in neurotransmitters have been identified, including acetylcholine, dopamine, glutamate, GABA, histamine, and noradrenaline, but none (not even acetylcholine, which is believed to have the strongest implication) has been consistently associated with every case [21]. This suggests that a wide variety of baseline characteristics and triggers can cause a similar syndrome, though certain mechanisms such as acetylcholine deficit may be more commonly involved. Ultimately, these mechanisms lead to a brain network dysfunction in which several structures are implicated in the symptoms of delirium, including the hippocampus, thalamus, basal forebrain, and cerebellum (and associated white matter tracts: fimbria, fornix, internal capsule, corpus callosum) [22].

The diagnosis of delirium is clinical and the Confusion Assessment Method (CAM) is the most commonly used and reliable screening tool in clinical practice [23]. Once the diagnosis has been confirmed, additional diagnostic tests such as laboratory testing, brain imaging, and electroencephalography (EEG) may be helpful in detecting potential treatable triggers. Traditionally, delirium was considered a transient condition that resolved after addressing the precipitating factors. Nonetheless, a growing body of evidence indicates that symptoms may persist for months in one third of patients [24, 25].

PD has been linked to a heightened risk of developing delirium; however, studies in this field vary considerably in the definition of delirium, in the methods employed for both delirium and PD diagnosis, and in the characteristics of included patients [26]. A recent prospective study, using a standardized diagnostic algorithm based on the DSM-5, estimated a delirium prevalence of 31% among inpatients with PD [27]. The susceptibility of PD patients to delirium seems to originate from a convergence of several overlapping mechanisms between the two conditions: a systemic inflammatory response, neurotransmitter imbalances (including disruptions in the cholinergic system), and alpha-synuclein pathology, which has been independently linked to postoperative delirium in gastrectomy samples [28]. Furthermore, delirium is correlated with an elevated risk of PD-CI, which in turn enhances the likelihood of delirium, highlighting its significance as a crucial interacting factor [29].

Clinical features of PD and delirium exhibit similarities, including attentional dysfunction, cognitive fluctuations, hallucinations, sleep disturbances, and excessive daytime sleepiness, which are commonly found in both syndromes [26]. It should be noted that assessment tools to detect delirium, such as CAM, have not been validated for use in PD, potentially leading to the misdiagnosis of delirium with long-standing PD symptoms, particularly among non-specialists.

### Non-motor cognitive and neuropsychiatric fluctuations

NMF typically accompany motor fluctuations but may also occur during the early stages of disease when motor fluctuations are not yet evident [30]. Although NMF are not as extensively documented as motor fluctuations, they can be equally or even more detrimental.

Cognitive fluctuations are a subtype of NMF that typically emerge during the ‘off’ state [31]. These symptoms are difficult to assess and measure, often being reported as a subjective mental slowness or difficulties in concentration [31]. Recent studies have identified that up to 23.1% of patients with PD experience cognitive NMF (assessed using the MDS Non-Motor Rating Scale) [30]. Its prevalence increases as the disease progresses, with the highest prevalence in patients with a disease duration > 10 years [32]. Occasionally, individuals may experience ‘on’ period bradyphrenia and difficulties in memory retrieval, which could also be attributed to the cognitive side effects of L-dopa, as discussed below [33].

Neuropsychiatric symptoms, such as hallucinations, may also manifest as fluctuations in PD, usually associated with the ‘on’ state but also reported during ‘off’ periods [33].

### Cognitive side effects of PD medications

In nearly all PD patients, dopamine-replacement therapy with L-dopa or other dopaminergic medications is required within the first few years after diagnosis [34]. Dopaminergic medications can affect cognition in both healthy and PD individuals, with different effects depending on the baseline dopamine levels, speed of dopamine level increase, and cognitive tasks used for evaluation [35–40]. These effects are caused not only by the impact of L-dopa on the dopaminergic system, but also on the serotoninergic and noradrenergic systems, which are associated with cognitive and neuropsychiatric symptoms in PD, and which may be influenced by L-dopa due to the aminergic characteristics of their cells [41, 42].

Most classical studies that evaluated the cognitive effects of dopaminergic stimulation used L-dopa [43]. It has been demonstrated that the dopamine–cognition relationship does not follow a linear function; instead, it generally follows an “inverted-U” function. This means that the dose of dopamine can improve cognitive performance up to a certain point after which, despite further dose escalation, cognitive performance will decline [44, 45]. Two competing theories have been proposed to explain this pattern. (1) The “overdose hypothesis” is the most supported theory, which suggests that dopamine can enhance the performance of more degenerated structures like the dorsal striatum, but impairs the performance of less damaged structures like the ventral striatum due to a dopamine overdose [35, 39, 46]. (2) The “dopamine-denervation theory” predicts an opposite pattern, with greater impairment of more denervated structures after dopaminergic stimulation and less impairment of more conserved structures [47]. Nevertheless, the cognitive effects of L-dopa have predominantly been studied in the short-term; its long-term effects remain less understood.

Research on the cognitive effects of dopaminergic agonists have yielded contradictory results. However, they are believed to follow a similar “inverted-U-shape” cognitive pattern as dopaminergic stimulation, and are also associated with a higher potential for hallucinations [48–50]. Other drugs commonly used in the advanced stages of PD, such as anticholinergics, amantadine or selegiline, may also induce cognitive impairment or hallucinations [51, 52].

### PD-associated psychosis

Psychotic symptoms, especially visual hallucinations, are common in PD, with a prevalence ranging from 16 to 75% [28]. The diagnosis of PD-associated psychosis requires a confirmed PD diagnosis and the presence of at least one psychotic symptom (illusions, hallucinations, delusions, or false sense of presence) that has recurred or persisted for 1 month after the onset of PD [53]. The diagnosis should also rule out delirium, delusional disorders, psychiatric disorders, or other conditions like DLB [53].

Several factors, including disease-related factors, medications, or intercurrent illnesses, may trigger or exacerbate psychosis in PD. However, these symptoms can also appear even without their influence, suggesting that it is a disease-specific phenomenon [28]. The most significant disease-related factors include PD progression and CI. The latter, especially in the PDD stage, is strongly associated with an increased risk of psychosis and more intractable symptoms [54, 55]. Individuals without PD-CI may also experience visual hallucinations, suggesting the presence of other contributing disease-related factors [41]. Medications, especially dopaminergic therapies, can be a trigger for psychosis—but it can also occur without the influence of drugs, suggesting that it is not solely a drug-related disorder [56–58]. Intercurrent medical illnesses are among the most common causes of developing psychotic symptoms in individuals with PD. It is essential to differentiate these psychotic symptoms from delirium, which usually presents with a more sudden onset, impaired attention, consciousness, orientation, and logical thinking, and is often related to treatable secondary causes [28].

The pathophysiology of PD-associated psychosis is thought to involve the dopaminergic system, as well as the cholinergic, glutamatergic, and serotonergic systems [53]. Neurodegeneration in the temporo-limbic and neocortical prefrontal areas is considered particularly significant, as it impairs processes such as visual processing, reality perception, and attention [28]. These changes share similarities with the pathophysiology of delirium.

## Diagnostic and therapeutic challenges and caveats

The main challenge in individuals presenting with cognitive disorders in PD is to determine whether an underlying secondary illness requires specific treatment (delirium) or symptoms result purely from PD progression or antiparkinsonian medications. The symptomatic treatment for cognitive or behavioral symptoms may also differ, depending on the scenario. This is difficult because there are numerous overlapping similarities between delirium and PD symptoms, including attentional and cognitive fluctuations, hallucinations, and sleeping disorders, and there is no screening tool that has been specifically validated for PD. Furthermore, delirium may also concur with PD-associated cognitive and behavioral symptoms. With regard to the temporal course of the symptoms detailed medical history of care-givers may be essential.

### PD-CI versus delirium

The majority of individuals with PD will develop PD-CI over the course of the disease [59]. CI serves as a major predisposing factor for delirium in the general population, particularly in neurodegenerative disorders; at the same time, several studies have reported an increased risk of developing PD-CI years after the first hospital episode of delirium, regardless of the underlying trigger or the baseline cognitive level [27, 29, 60]. Furthermore, delirium may result in incomplete recovery, with persistent CI [4, 20, 61]. This bidirectional interaction and the frequency of PD-CI creates a common scenario in which patients experience acute delirium alongside concurrent baseline PD-CI, or delirium followed by immediate or delayed PD-CI.

The clinical similarities between delirium and PD-CI pose challenges in distinguishing between the two when assessing a patient at a single point in time. To overcome this, temporal dynamics become a critical factor. An acute onset within hours to days and fluctuating symptoms suggest delirium, whereas a progressive, long-term, persistent and stable clinical course points towards PD-CI. The criteria defined by the MDS for PDD or PD-MCI do not specify a minimum time since symptom onset [8]; however, World Health Organization criteria for dementia require a minimum course of 6 months. Yet distinguishing persistent delirium from PD-CI progression can be challenging. The brain consequences of delirium might potentiate PD-CI, but quantifying the contribution of each condition to the overall deterioration remains difficult. Another crucial clinical factor is a clear decline from the baseline cognitive status. While this information proves valuable in the initial stages of PD-CI, the diagnosis in patients with more severe PD-CI is more difficult. Ultimately, PD-CI is considered an exclusion diagnosis [7, 8].

Rivastigmine is the only approved drug for PDD, while no drug has received official approval for PD-MCI [62, 63]. Although the clinical effect on cognition is modest, rivastigmine can reduce the frequency of hallucinations [64, 65]. At present, rivastigmine or other cholinesterase inhibitors are not approved for delirium in the context of PDD, and its potential benefits remain unclear. Controlled studies on the treatment of delirium with rivastigmine in PD are lacking. In cases where individuals with PD-CI experience predominating psychotic symptoms, the addition of neuroleptic drugs such as quetiapine or clozapine is recommended [66]. In the case of clozapine, however, the anticholinergic side effects potentially promoting delirium need to be carefully balanced.

### Non-motor cognitive and neuropsychiatric fluctuations versus delirium

Cognitive NMF can manifest with an acute onset inattention and fluctuating arousal, typically occurring when the effect of L-dopa disappears. Fluctuations tend to increase over the course of the disease, implying that patients with a higher NMF burden will probably have more severe PD-CI and more predisposing factors for delirium [32]. Additionally, severe motor “off” states or NMS such as unnoticed orthostatic hypotension may also mimic symptoms of delirium.

The management of NMF consists of optimizing antiparkinsonian medications to achieve stable dopaminergic stimulation of the striatum. Assessing the response to medication can be useful in differentiating NMF from hypoactive delirium [67]. However, akin to all cognitive disorders in PD, cognitive NMF may overlap with delirium depending on the context; according to DSM-5, certain NMF symptoms may meet criteria for the diagnosis of delirium secondary to medication withdrawal.

### Cognitive side effects of PD medications versus delirium

Similarly to NMF, the cognitive side effects of PD-related medication, non-PD-related medication or multiple drug interactions may in certain cases promote factors of delirium. In these cases, delirium secondary to drugs may be diagnosed according to DSM-5 criteria [18]. The management of drug-related cognitive side effects in PD typically requires discontinuation of drugs according to their delirogenic potential and a reduction in the overall dosage of dopaminergics, particularly when symptoms are prolonged and significantly disruptive [68]. This may entail a deliberate worsening of Parkinsonian motor symptoms. Anticholinergics, amantadine, or dopamine agonists should be discontinued first before reducing levodopa, which offers the most favorable ratio between motor symptoms improvement and the likelihood of cognitive adverse effects. Additionally, the administration of neuroleptics may be necessary when psychotic symptoms are present [68].

### Parkinson-associated psychosis versus delirium

Visual hallucinations are common in delirium, although not necessary for its diagnosis. They are also a common feature of PD, even in early disease stages, and are a typical but not exclusive feature of PD-associated psychosis.

From a pragmatic perspective, a subacute worsening of hallucinations in individuals with PD, in particular when accompanied by other symptoms such as loss of insight, cognitive fluctuations, attentional disturbances, and other acute cognitive impairments, could justify a clinical workup for common treatable causes of delirium. When these hallucinations persist and remain stable for more than 1 month, they are considered a natural part of the disease and not related to external insults [53]. Nevertheless, it is likely that delirium and PD-associated psychosis often form part of the same spectrum (Table 3).

**Table 3:** Summary of the different features of cognitive disorders in Parkinson’s disease

	NMF (off)	NMF (on)	Delirium	PDD	Psychosis
Disturbance of alertness	+	+	+++	+	
Disturbance of vigilance	+		+++	++	
Disturbance of orientation	+	+	+++	++	
Hallucinations	+	+	++	+	+++
Delusions	+	+	++	+	+++
Fluctuations	++	+++	++	+	
Acute onset	+	++	+		+

NMF, Non-motor fluctuations; PDD, Parkinson’s disease dementia; +, Occasional; ++, Often; +++, Usually

Management includes ruling out secondary causes and attempting to reduce dopaminergic medication; if this approach is insufficient or the symptoms are highly intense from the outset, the addition of neuroleptic drugs is recommended [69–73].

## Management of delirium in PD

### Diagnosis of delirium

The diagnosis of delirium continues to rely on clinical criteria, and an objective biomarker has not yet been identified [74, 75]. Similar to management in the general population, when delirium is diagnosed or suspected, additional tests and examinations should be performed to detect triggering factors, as it may be the sole clinical indicator of a life-threatening condition. Routine laboratory tests, including urine analysis, are usually included in the initial evaluation, while brain imaging and cerebrospinal fluid (CSF) testing are only performed in selected patients based on clinical history. Other causes of cognitive impairment or hallucinations in PD should always be considered.

### Utility of additional diagnostic tools

#### Neuropsychological assessment

The prototypical neuropsychological profile in delirium is characterized by attention deficit with fluctuating features, often accompanied by arousal impairment [18]. When patients are awake, a wide range of disturbances in other cognitive domains may also be detected, such as memory deficit, disorientation, language impairment, visuospatial ability, or altered perception. However, these disturbances are not specific and cannot be relied upon to distinguish delirium from dementia syndrome [74].

Numerous delirium assessment scales are described in the literature. A review of 24 scales identified the CAM [76], Delirium Rating Scale [77], Memorial Delirium Assessment Scale (MDAS) [78], and NEECHAM Confusion Scale [79] as the most reliable screening tools [80]. Currently, there are no validated methods to diagnose delirium in established LBD [81]. In a recent study, Lawson et al. [82] identified the most sensitive bedside tests for assessing attention and arousal to detect delirium in LBD: attention—the backwards digit-span, months of the year backwards test, and MDAS attention item; arousal—Glasgow Coma Scale, Observational Scale of Level of Arousal, Modified Richmond Agitation Scale (m-RASS), and MDAS consciousness item.

#### EEG

Multiple studies have attempted to detect specific alterations in EEG for delirium or PD-CI. Some typical patterns have been identified, such as a reduction or loss of the posterior rhythm, generalized slowing, and intermittent deltoid activity in delirium [83], or slowing of the EEG in LBD [84]. However, posterior slowing of alpha rhythm also can be found in PD, PDD and LBD, and is not specific for delirium. The main utility of EEG in routine clinical practice is for excluding nonconvulsive or subclinical seizures or for guiding the diagnosis of certain metabolic encephalopathies or infectious encephalitides with characteristic EEG patterns.

#### Laboratory tests

Selected laboratory tests should be conducted to rule out secondary causes, depending on the clinical history. However, there is no reliable laboratory parameter in serum or CSF that can be used to detect the risk or diagnose delirium. Some studies have identified altered parameters associated with delirium, such as an increased level of C-reactive protein in serum or a reduced concentration of beta-amyloid 1–40 in CSF [85]. These findings are believed to reflect predisposing or triggering factors rather than specific biological processes related to delirium.

#### Positron emission tomography

There is mounting evidence of disrupted cerebral metabolism in delirium. A prospective study investigating 2-18F-fluoro-2-deoxyglucose positron emission tomography (PET) patterns of cerebral glucose metabolism in older hospitalized patients with delirium revealed widespread and reversible cortical hypometabolism [86]. Nonetheless, these studies are not yet adequate nor specific enough to establish PET as a useful tool in clinical practice, and further research is necessary.

### Treatment of delirium in PD

No studies have evaluated specific interventions to prevent or treat delirium in PD and current guidelines are primarily based on clinical experience [26, 87, 88]. The initial and most important goal is to treat any precipitating underlying illness, especially bacterial infections. Metabolic alterations (hypo-/hyperglycemia, hypoproteinemia, uremia, electrolyte dysturbances, etc.) have to be compensated. Medical therapy should follow the recommendations of drug therapies in the elderly and avoid polypharmacy and potentially inadaequate medications, especially those with anticholinergic activity (Table 4). Withdrawal of preexisting inadaequate medications can be considered, but abrupt withdrawal should be avoided.

**Table 4:** Recommendations concerning pharmocological treatments in PD patients with delirium

Indication/symptoms	To be avoided	To be prefered
Infections (pneumonia, cystitis …)	Fluorchinolons, Nitrofurantoin	ß-lactam antibiotics, makrolids
pain	Opioids	Ibuprofen, Paracetamol, Metamizol
Insomnia	Benzodiazepines, z-substances, H1-antihistamines	Melatonin, Quetiapine, Mirtazapine
Incontinence	Oxybutinin, Solifenacin, Tolterodine	Mirabegron, Trospiumchloride
Antidepressants	Tricyclic antidepressants	SSRI, Mirtazapine
PD symptoms	Anticholinergics Amantadine Dopaminagonists MAO-B-inhibitors Opicapone (withdrawal in this order)	L-dopa, Entacapone
Neuroleptics	Typical neuroleptics	Quetiapine
Antidementives	Avoid acute withdrawal or escalation of dosage	

Pharmacological management of delirium in PD presents particular challenges: dopaminergic drugs may exacerbate delirium, withdrawal of these drugs may also induce delirium, and the main medication for delirium in general population (neuroleptics) can worsen parkinsonism. There is no reasonable evidence-based approach targeting hypoactive delirium. In hyperactive delirium agitation, fear, hallucinations and/or delusions may need pharmacological intervention. If necessary, quetiapine is considered the safest option for PD, although controlled studies on its use in PD-related delirium are lacking [87]. Quetiapine usually is administered in dosages from 12.5 to 150 mg per day and efficacy may be insufficient. Clozapine is effective in the treatment of hallucinations and delusions, but evidence in PD is limited to the treatment of psychosis apart from the context of delirium (dosage: 6.25–100 mg per day). Clozapine has anticholinergic action and may itself induce delirium, especially in combination with benzodiazepines. Some experts favour low-dose risperidone (max. 1 mg per day) in PD-related delirium taking into account the extrapyramidal side effects. Conversely, discontinuing or reducing the dosage of antiparkinsonian drugs should also be considered.

General measures to prevent and treat delirium encompass the design of a low-stimulus environment, establishing an adequate day-night structure, providing access to aids such as glasses, hearing aids, and calendars, rooming-in or involvement of family members in the care process, promoting patient mobilization, and reducing stressors such as pain, hunger, and thirst [89]. However, it is yet to be determined whether the preventive measures proven effective in the broader older adult population have the same impact on individuals with PD.

## Conclusions

Cognitive disorders in PD encompass various syndromes: PD-CI, delirium, cognitive side effects of PD medications, cognitive NMF, and PD-associated psychosis. Broadly, these can be categorized into those primarily caused by the disease and those that require additional external factors to occur. The importance of distinguishing delirium from other cognitive or behavioral disorders in PD lies in the fact that delirium requires specific etiological treatment, while for the latter only symptomatic treatment options are available. Currently, there is no recognized gold standard for diagnosing delirium, and diagnosis relies on clinical evaluation. Moreover, none of the clinical scales used in the general population have been validated in individuals with PD.

Diagnosis may be straightforward in the early stages of the disease, but as the disease progresses and baseline cognitive symptoms become more evident, this task becomes more challenging. There is a bidirectional relationship between delirium and PD-CI, where delirium is a risk factor for PD-CI and vice versa. Hence, a higher percentage of PD patients experiencing delirium will have CI, further complicating the diagnostic process.

Additionally, there is a clinical overlap and synergy between delirium and PD. They both share common symptoms, making it challenging to differentiate between delirium and other cognitive and behavioral disorders in PD. There is no clear demarcation between the used terms for these disorders, and ultimately, it is difficult to establish precisely when PD-CI ends and delirium begins. Similar mechanisms are involved in the pathophysiology of PD and delirium, suggesting that they may be part of a spectrum. A similar concept has already been embraced in psychiatry, where the differentiation between endogenous and reactive depression has been abandoned, and individual factors are integrated within a biopsychosocial model [90]. The pragmatic consequence for daily clinical practice would be to suspect a partial delirium component in every PD patient with a cognitive disorder, and to consistently apply the recommended diagnostic and general measures for delirium to minimize this component as much as possible.

In summary, beyond an appropriate terminology by which we try to categorize a spectrum of cognitive disorders in PD, it is clinically essential to identify delirium as the only form of PD-CI which may be fully reversible when managed appropriately. Future research should focus on identifying specific biomarkers for delirium applicable to PD, and validating clinical scales that can be utilized in clinical practice by non-specialist physicians.

## Data Availability

Not applicable.

## Abbreviations

CAM: Confusion Assessment Method
CI: Cognitive impairment
CSF: Cerebrospinal fluid
DLB: Dementia with Lewy bodies
DSM-5: Diagnostic and Statistical Manual of Mental Disorders
EEG: Electroencephalography
MDAS: Memorial Delirium Assessment Scale
MDS: Movement Disorders Society
MMSE: Mini-Mental State Examination
NMF: Non-motor fluctuations
NMS: Non-motor symptoms
PD: Parkinson’s disease
PD-CI: Parkinson’s disease-associated progressive cognitive impairment
PDD: Parkinson’s disease dementia
PD-MCI: Parkinson’s disease–mild cognitive impairment
PET: Positron emission tomography
SCD: Subjective cognitive decline
